# Effects of Ag and melt undercooling on the microstructure of Sn–Ag solder balls

**DOI:** 10.1007/s10854-025-14979-6

**Published:** 2025-06-02

**Authors:** Sihan Sun, Ao Li, Chao Cheng, Christopher M. Gourlay

**Affiliations:** Department of Materials, Imperial College London, London, SW7 2AZ UK

## Abstract

The microstructure of electronic solder joints is created by solidification in an undercooled melt. Here, we apply the framework of solidification microstructure selection maps (SMSMs) to Sn-Ag solder balls with compositions from 0.5 to 5.0 wt.% Ag and bulk undercoolings in the range 10–70 K. The effects of Ag and melt undercooling on the transition from single-grain to cyclic-twinned microstructures and the extent of interlacing are revealed. Controlled by the Ag content and the competitive nucleation between the β-Sn and Ag_3_Sn phases, a competition between tin dendrite and eutectic growth is observed that decides the microstructure of solder balls. The solidification microstructure selection map, modified to account for whether Ag_3_Sn or β-Sn nucleate first in competitive nucleation, is in reasonable agreement with coupled zone theory. The map provides a guide for tailoring desired solder microstructures for mechanical performance through controlling the nucleation undercooling of β-Sn and Ag_3_Sn during solidification.

**Supplementary Information:**

The online version contains supplementary material available at 10.1007/s10854-025-14979-6.

## Introduction

The microstructure of electronic solder joints plays an important role in solder joint reliability [1, 2] and forms by the solidification of a droplet of undercooled liquid. In the case of Sn–Ag and Sn–Ag–Cu alloys in ball grid array (BGA) soldering, there is usually a single nucleation event, producing either a single β-Sn grain or three orientations interrelated by cyclic twinning [3–8]. The cyclic-twinned microstructures have been found to have either a beachball morphology, an interlaced morphology, or a mixture of the two (i.e. partially interlaced morphology). In the literature, beachball microstructures vary from (i) those that closely resemble a beachball with six near-equal segments and near-straight {101} boundaries (e.g. [5, 8, 9]) to (ii) more irregular microstructures of three cyclic-twinned orientations without interlacing ([10–12]). Interlaced microstructures appear to have a small grain size but usually still only consist of three main orientations interrelated in the same way as beachball grains [5, 6, 13].

Lehman et al. [5] studied the crystallographic aspects of β-Sn cyclic twinning in detail and proposed that it originates at the nucleation stage by a nucleus with hexagonal symmetry around a central Ag atom. Arfaei et al. [6] deduced that lower solidification temperatures (i.e. more deeply undercooled melts) result in a higher degree of interlacing, although the undercooling—interlacing relationship was not directly measured. Now there is a need for a systematic study of the combined roles of Ag and melt undercooling on the solidification microstructure including β-Sn cyclic twinning, and the conditions for producing fully eutectic microstructures. This can be partly achieved through the development of a solidification microstructure selection map (SMSM).

SMSMs are graphical representations of the phases and microstructures that solidify under some combination of alloy composition (*C*_0_) and solidification conditions such as temperature gradient (*G*), interface velocity (*V*) or melt undercooling (Δ*T*) [14–16]. SMSMs have been applied commonly to solidification into a positive temperature gradient [16], including during Bridgman growth [14, 17], laser remelting [18] and, more recently, additive manufacturing [19, 20]. There, they are usually plotted as a *V*-*C*_0_ map at a fixed temperature gradient or a *G*-*V* map for a given alloy composition. In Sn-Ag alloys, solidification studies in a positive temperature gradient [21–25] have captured how the microstructural length scale depends on *V*, *G* [24, 26] and cooling rate [27], and generated *V*-*C*_0_ maps that showed the eutectic coupled zone is skewed to the Ag_3_Sn side [23, 25].

SMSMs have also been applied to the solidification of bulk undercooled melts [28–31], typically plotting phases and microstructures on a *C*_0_ versus Δ*T* map [29, 30] or on a *C*_0_ versus temperature (*T*) phase diagram [28, 31–33]. Here, the plotted undercooling or temperature usually refers to the moment of nucleation which is then followed by recalescence [34]. While studies have focused on various aspects of solidification in undercooled Sn-Ag droplets [5, 35–39], *C*_0_-*T* maps have not been developed for Sn-Ag alloys previously. Such *C*_0_-*T* maps are particularly relevant to ball grid array soldering where solidification is predominantly nucleation controlled.

In this paper, we study the effects of Ag concentration and melt undercooling on the microstructure of ~ 500 μm diameter freestanding solder balls cooled at 0.33 K s^−1^ in a differential scanning calorimeter (DSC). We first investigate the distribution of nucleation undercoolings at different Ag concentrations. We next focus on the role of Ag and undercooling on cyclic twinning, the extent of interlacing, and the formation of β-Sn dendrites, Ag_3_Sn plates and eutectic. We then develop a SMSM for Sn-xAg compositions from 0.5 to 5.0 wt.% Ag solidified at bulk undercoolings in the range 10–70 K, so that hypo-eutectic, near-eutectic and hyper-eutectic compositions are all covered, and explore the results within the frameworks of coupled zone theory and rapid solidification.

## Methods

Sn-3.5Ag and Sn-5Ag (wt.%) were made by mixing 99.999% Sn with 99.9% Ag in a graphite crucible, heating to 300 °C and holding for 2 h in a resistance furnace. The alloy melt was then stirred with an Al_2_O_3_ rod and poured into a steel mould. Some of the Sn-5Ag was then used as a master alloy and mixed with 99.999% Sn again to make Sn-0.5Ag and Sn-2Ag with the same procedure. The ingots were then rolled into foils with thickness of about 30 µm and a 1.5 mm diameter punch was used to produce solder disks. Next, they were remelted with a ROL1 flux (IPC J-STD-004) on a hot plate at 270 °C to form solder balls of approximately 500 µm diameter under surface tension. Finally, at least 10 min of ultrasonic treatment in an ethanol bath was applied to remove the flux.

**Fig. 1:**
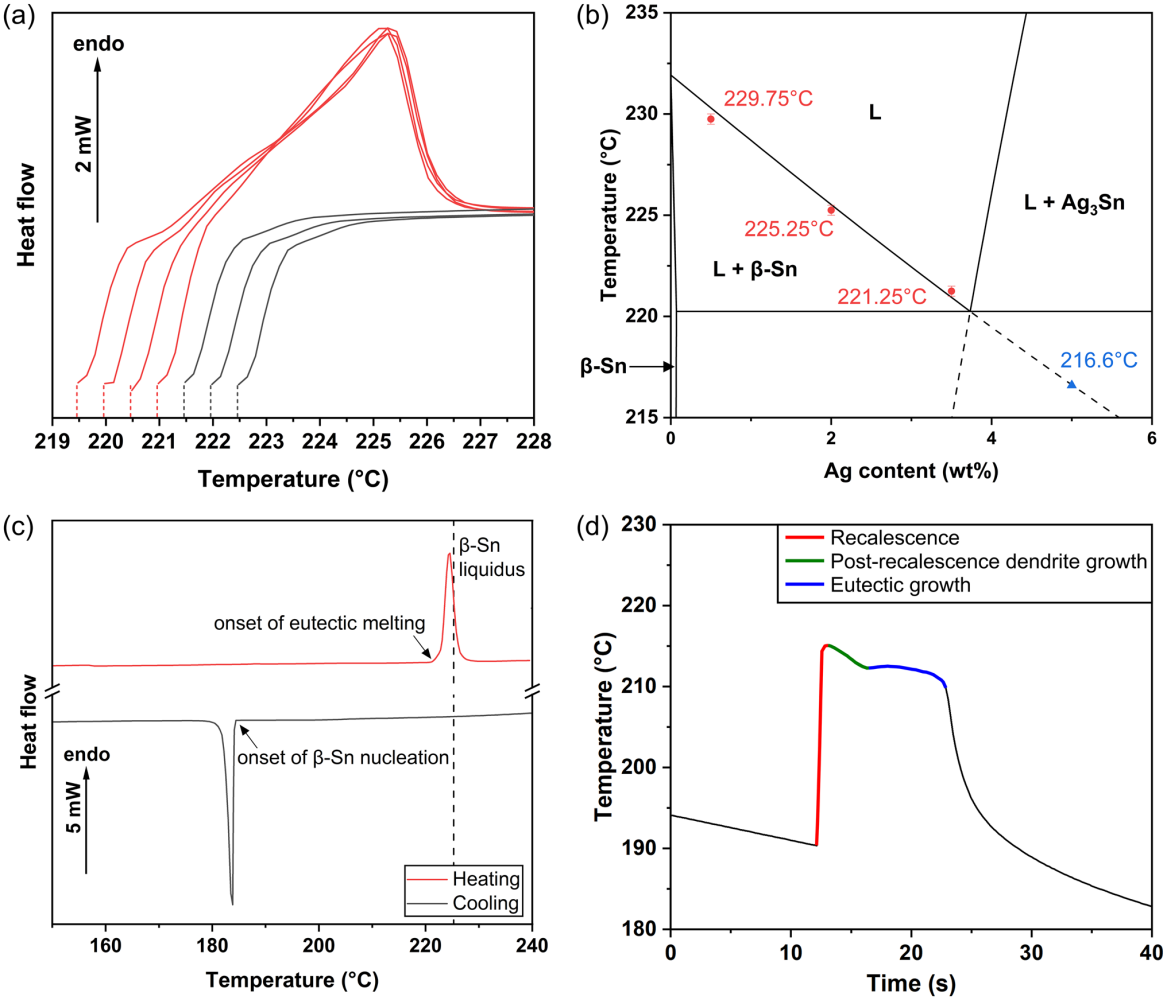
DSC experiments. **a** Cyclic DSC method to measure the β-Sn liquidus temperature. Dashed vertical lines indicate prior holding temperatures, for 30 min each. The endothermic peak in the red curves indicates that β-Sn was not fully melted at these prior holding temperatures. The lack of an endothermic peak in the black curves indicates that β-Sn was fully melted at these prior holding temperatures. The β-Sn liquidus temperature is 221.25 ± 0.25 °C in this example. **b** Sn–Ag phase diagram from the Thermo-Calc TCSLD4.1 database with the measured β-Sn liquidus temperatures in red. **c** Typical DSC experiment to measure β-Sn nucleation temperature on 500 *μ*m balls. **d** Cooling curve of solder ball solidification using a thermocouple immersed in a larger ball in the DSC, demonstrating the three stages of solidification in a hypoeutectic ball

Solidification experiments were performed in a DSC (Mettler Toledo DSC-1) in aluminum pans under a nitrogen atmosphere. First, to measure the β-Sn liquidus temperature for each Sn-Ag composition, a cyclic DSC method similar to that in references [40–43] was applied as summarised in Fig. 1a and its caption. Figure 1b plots the measured β-Sn liquidus temperatures for Sn-0.5Ag, Sn-2Ag and Sn-3.5Ag in red on the Sn-Ag phase diagram from the Thermo-Calc TCSLD 4.1 database, showing good agreement. For Sn-5Ag, the β-Sn liquidus temperature could not be measured by this method because of its hypereutectic composition and, thus, its metastable β-Sn liquidus temperature was taken from the TCSLD 4.1 database and is plotted as a blue symbol.

Next, DSC was used to impose controlled cooling conditions to solder balls during solidification and measure the β-Sn nucleation temperature. The thermal profile involved heating at 20 K/min to 270 °C for Sn-5Ag and 250 °C for the other compositions, followed by cooling at 20 K/min. Figure 1c shows a typical DSC profile, from which the β-Sn nucleation temperature is defined as the onset of the exothermic peak on cooling. The β-Sn nucleation undercooling in this study was calculated by subtracting the β-Sn nucleation temperature from the β-Sn liquidus temperature. To accelerate throughput, a Mettler Toledo sample robot was used to sequentially load 34 pans containing one solder ball each into the DSC. For each composition, at least 240 balls were solidified individually in the DSC which resulted in β-Sn nucleation undercoolings in the range of ~ 10–70 K.

For each composition, the solder balls were split into different bins based on their β-Sn nucleation undercooling, using a bin width of 5 K. Multiple (up to 30) solder balls within the same undercooling bin were mounted together with Struers VersoCit-2 acrylic resin. Then the mounted samples were ground with water on SiC papers of increasing grit numbers until 4,000 grit, before polishing with a mixture of 50 vol% water and 50 vol% OP-S non-drying colloidal silica suspension (Struers) for 10 min. Finally, 5 min of ultrasonic bathing in water was applied to wash away the OP-S.

An Olympus BX53M optical microscope equipped with polarizing filters was used to capture optical micrographs of Sn-Ag solder balls. A Zeiss Sigma 300 field emission gun scanning electron microscope (FEG-SEM) installed with a Bruker eFlash HR electron backscatter diffraction (EBSD) detector was used to map the β-Sn grain orientations. A Zeiss Auriga FEG-SEM was used to obtain backscattered electron micrographs. Bruker Esprit 2.1 software was used to process the EBSD data.

Note that the DSC method in Fig. 1c only measures the nucleation temperature and does not measure the temperature of the ball as it solidifies. One additional experiment was performed on a larger hypoeutectic solder ball (~ 1.5 mm diameter) where an 80 μm diameter K-type thermocouple was immersed in a liquid solder ball on a hotplate. This ball and thermocouple were then fixed in the DSC chamber with polyimide tape and resolidified using the same thermal profile as the main study in Fig. 1c. As shown in Fig. 1d, this immersed thermocouple method captures the recalescence and the three stages of solidification in a hypoeutectic solder ball after nucleation in an undercooled melt.

## Results and discussion

### Effects of Ag and bulk undercooling on microstructure

#### Summary of microstructure types

The microstructures that solidified in the Sn–Ag balls depended on the alloy composition, the undercooling for β-Sn nucleation and whether Ag_3_Sn nucleated before β-Sn or not. In some cases, there were clear differences in the microstructure that formed during recalescence and that which grew after. These microstructural features can be loosely classified as: (i) the primary β-Sn grain morphologies reported previously for Sn-Ag and Sn–Ag–Cu balls [6, 44, 45]: single-grain, cyclic-twinned beachball, partially interlaced and fully interlaced microstructures; (ii) Solder balls with regions of very fine β-Sn dendrites; and (iii) Solder balls with large fully eutectic regions.

In sections 3.1.2 and 3.1.3, these features are presented separately. Then the results are combined into a microstructure selection map and discussed with the help of equations for competitive eutectic and dendrite growth in section 3.2. In Sn-5Ag samples, balls where Ag_3_Sn nucleated first and grew to a large size before β-Sn nucleated went on to solidify with primary β-Sn grain morphologies, whereas balls where β-Sn nucleated first solidified with large fully eutectic regions.

#### Effects of Ag and bulk undercooling on primary β-Sn grain morphologies

**Fig. 2:**
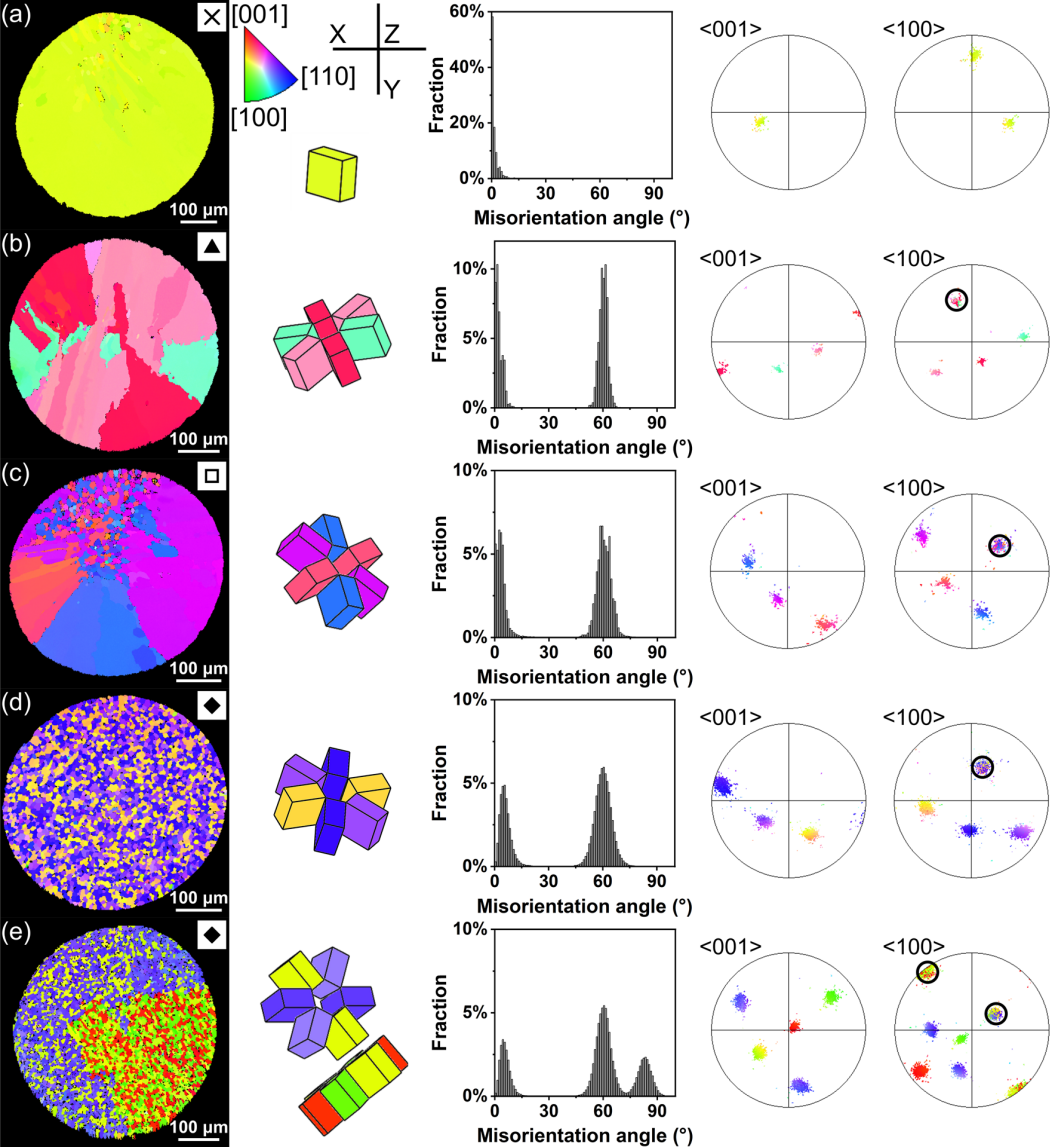
EBSD orientation maps of four typical β-Sn grain morphologies: **a** single-grain, **b** beachball, **c** partially interlaced and **d**, **e** fully interlaced. **a**–**e** are all Sn–2Ag balls. For each sample, from left to right: the orientation (IPF) map, unit cell wireframes of the main β-Sn orientations using the same IPF colouring, the β-Sn misorientation distribution, and the {001} and {100} pole figures of β-Sn. Black circles in the pole figures highlight common axes shared by three orientations. **d** shows a fully interlaced sample with one cyclic twinning axis whereas **e** shows a sample with two interrelated twinning axes giving a double ring. The symbols on the top right of the IPF maps are used to represent these grain morphologies in Fig. 10

The common β-Sn grain morphologies reported in Sn–Ag and Sn–Ag–Cu solder balls in past work (single-grain, cyclic-twinned beachball, partially interlaced and fully interlaced) [3–6, 44, 45] were also observed here. Since EBSD mapping of such microstructures has been reported in detail previously [2, 5, 6, 45–48], here the four microstructure types are only briefly presented in Fig. 2 and its caption.

Figure 2e also presents a special case of the fully interlaced morphology where there are two interrelated rings of β-Sn cyclic twins in the solder ball, similar to some previous studies [2, 45, 47, 49]. From the unit cell wireframe orientations, the two rings share a common yellow β-Sn orientation, and the rotation axis is the yellow [100] for one ring and the yellow [010] for the other ring. As a result, there are five grains in the solder ball that are all interrelated. The double ring of cyclic twinning results in another peak near 83° in the β-Sn misorientation distribution histograms corresponding to the misorientation between grains in different rings (e.g. between the red and purple orientations in Fig. 2e).

**Fig. 3:**
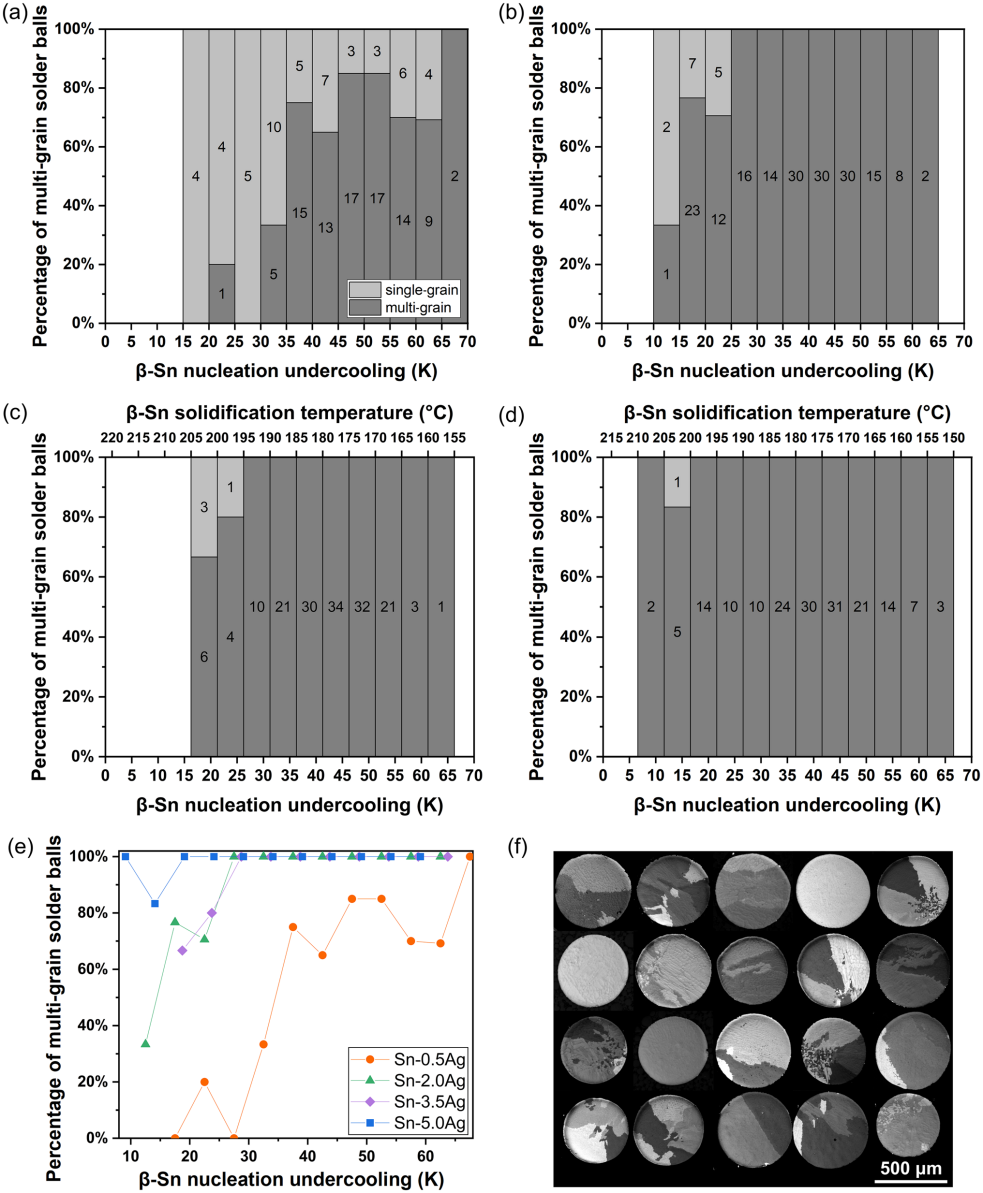
The effect of β-Sn nucleation undercooling on β-Sn cyclic twinning. **a**–**d** Column charts of the percentage of single-grain and multi-grain solder balls versus β-Sn nucleation undercooling for **a** Sn–0.5Ag, **b** Sn–2Ag, **c** Sn–3.5Ag and **d** Sn–5Ag. The number of balls in each bin is shown in the centre. **e** Scatter plot of the percentage of multi-grain solder balls versus β-Sn nucleation undercooling for the four compositions. **f** Polarized optical micrographs of 20 randomly chosen Sn-2Ag solder balls from the 15–20 K undercooling bin

Figure 3a–d exhibits the percentages of single-grain and multi-grain solder balls within each undercooling bin for the four compositions based on polarized light optical micrographs such as those in Fig. 3f. A clear trend was found in Sn-0.5Ag, Sn-2Ag and Sn-3.5Ag that solder balls with deeper β-Sn nucleation undercooling were more likely to have multiple β-Sn grains, in agreement with past work by Arfaei et al. [6] and Wang et al. [45]. For Sn-5Ag solder balls, such a trend could not be identified as only one single-grain solder ball was observed out of the 172 solder balls studied. Since EBSD mapping (e.g. Figure 2) showed that multi-grain microstructures were all cyclic-twinned, the results in Fig. 3 show that, for a given alloy composition, deeper nucleation undercooling promotes β-Sn cyclic twinning.

It was also found that, with increasing Ag content in the alloy, the Sn-Ag solder balls were more likely to be multi-grain and, therefore, cyclic-twinned. Figure 3e plots the percentage of multi-grain solder balls versus the β-Sn nucleation undercooling, combining the data from Fig. 3a–d. For a given undercooling bin, the percentage of multi-grain microstructures in Sn-0.5Ag balls was significantly smaller compared to the other three compositions, and there is a general trend that an increased Ag content in the alloy promotes β-Sn cyclic twinning.

A further result in Fig. 3 is that the transition from single-grain to cyclic-twinned microstructures does not occur abruptly at a critical undercooling or Ag content but occurs gradually such that deepening the undercooling or increasing the Ag content increases the probability of forming a cyclic-twinned microstructure.

**Fig. 4:**
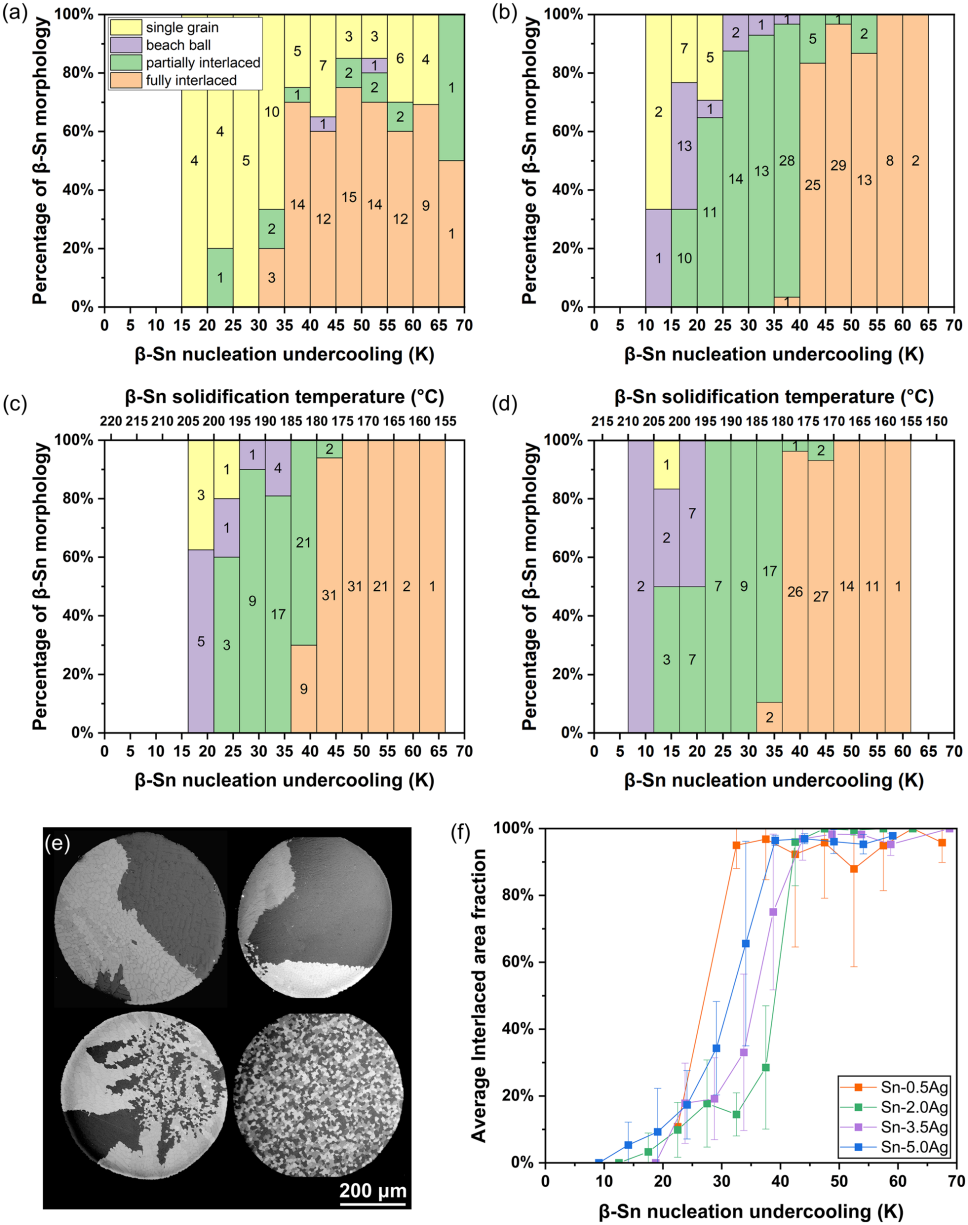
The effect of β-Sn nucleation undercooling on the β-Sn grain morphology. **a**–**d** Column charts of the percentage of solder balls with different grain morphologies versus β-Sn nucleation undercooling for **a** Sn–0.5Ag, **b** Sn–2Ag, **c** Sn–3.5Ag and **d** Sn–5Ag. The number of solder balls in each column are shown in the centre. **e** Polarized optical micrographs of cyclic-twinned Sn-2Ag solder balls showing different degrees of interlacing. **f** Scatter plot of average interlaced area fraction versus β-Sn nucleation undercooling in cyclic-twinned balls for the four compositions (excluding single-grain balls). The standard deviation of the data are shown as error bars

Figure 4a–d exhibit the percentage of solder balls with different β-Sn grain morphologies within each undercooling bin for the four compositions, adding further detail compared with Fig. 3. For Sn-2Ag, Sn-3.5Ag and Sn-5Ag, as the β-Sn nucleation undercooling became deeper, there was a gradual transition from single-grain to beachball, partially interlaced and, finally, fully interlaced microstructures. For Sn-0.5Ag, the morphology path is less clear because most of the solder balls were either single-grain or fully interlaced. Note that out of all the balls studied, four Sn-3.5Ag balls had microstructures that cannot be categorised convincingly to any of the four β-Sn grain morphologies. These balls are not included in Fig. 4 and their micrographs are given in the Online Resource 1. For the same reason, the Sn-5Ag solder balls with large fully eutectic regions are not included in Fig. 4. They are discussed in detail in Section 3.1.3.

To quantitatively study the development of interlaced microstructures with relation to β-Sn nucleation undercooling, the area fraction of interlacing was measured for all the solder balls. For example, Fig. 4e shows polarized optical micrographs of four Sn-2Ag solder balls with interlacing fraction spanning from 0% in the top left example to 100% in the bottom right example. The single-grain solder balls were excluded from the measurement as they were not regarded as having zero area fraction of interlacing in the present work. Figure 4f is a plot of the average interlaced area fraction versus β-Sn nucleation undercooling for each of the four compositions. All four compositions follow a similar path without any discernable effect of Ag content, indicating that the Ag composition does not significantly affect the degree of interlacing in solder balls that are cyclic-twinned, although the increased Ag content promotes the probability of β-Sn cyclic twinning occurring (Fig. 3e). It is also evident from Fig. 4f that the degree of interlacing in cyclic-twinned Sn-Ag solder balls increases with deeper undercooling and this occurs gradually over multiple undercooling bins. Note that, in fully interlaced solder balls, the measured area fraction of interlacing was often less than 100% because primary Ag_3_Sn occupied some of their cross sections.

**Fig. 5:**
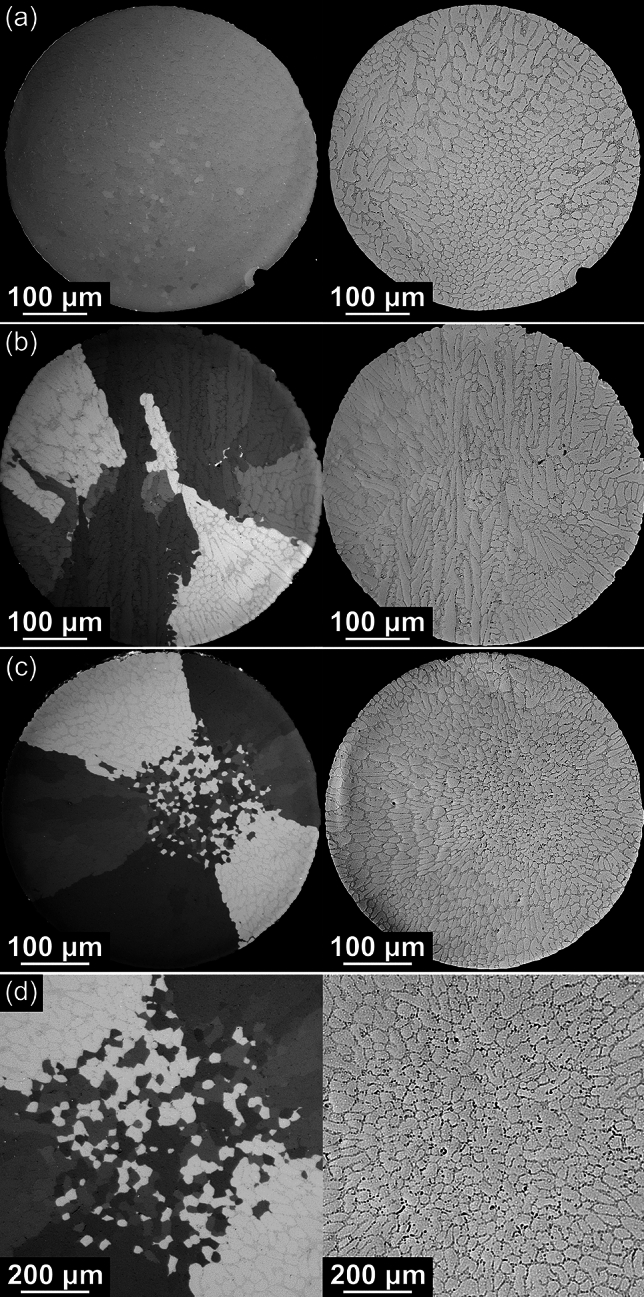
Polarized optical micrograph (left) and normal optical micrograph (right) of typical Sn–2Ag solder balls with **a** single-grain **b** beachball and **c** partially interlaced β-Sn morphologies. The small faint grains in the left image of **a** are subgrains with misorientation < 10° similar to ref. [50]. **d** Zoomed-in images of the interlaced region in (**c**)

All of the microstructure types in Figs. 2 and 4 contain primary β-Sn with eutectic β-Sn + Ag_3_Sn (and sometimes Ag_3_Sn plates). For example, Fig. 5a–c are typical microstructures of single-grain, beachball and partially interlaced solder balls of Sn-2Ag with both polarized and standard (non-polarized) optical micrographs. The optical micrographs confirm that single-grain and beachball regions consist of β-Sn dendrites with β-Sn + Ag_3_Sn eutectic between the dendrite arms. From Fig. 5d, the interlaced region consists of small primary β-Sn and Ag_3_Sn particles.

#### Solder balls with fully eutectic regions

**Fig. 6:**
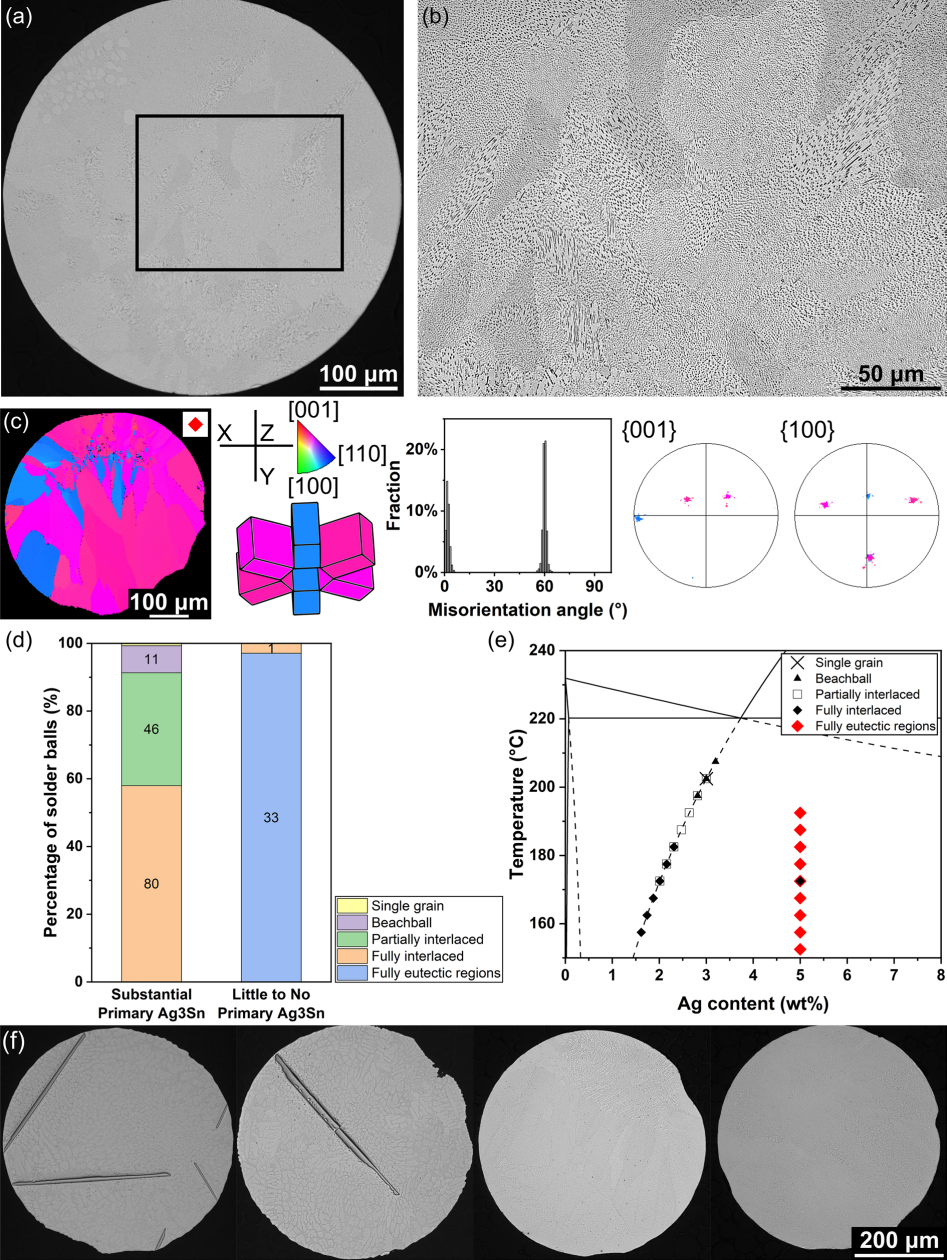
Overview of Sn-5Ag balls with large areas of fully eutectic microstructure. **a** Optical micrograph of a typical Sn–5Ag solder ball that does not contain primary Ag_3_Sn particles. **b** A zoomed-in optical micrograph of the area indicated by the black rectangle in (**a**). The entire area contains only eutectic microstructure. **c** EBSD analysis of a typical Sn–5Ag solder ball with large fully eutectic regions. From left to right: IPF-Z orientation map, β-Sn unit cell wireframe orientations for the main cyclic-twinned orientations, the β-Sn misorientation distribution, and the {001} and {100} pole figures of β-Sn. **d** Column chart of the percentage of different microstructure types in Sn-5Ag solder balls depending on the existence of primary Ag_3_Sn. **e** Microstructure selection map for Sn–5Ag on the Sn–Ag phase diagram. The data points of solder balls with substantial primary Ag_3_Sn have been moved onto the extended Ag_3_Sn liquidus line to indicate the changed liquid composition before β-Sn nucleates. Up to three symbols overlap at some points because different balls within an undercooling bin had different microstructures, as shown in Fig. 4. **f** Optical images of Sn–5Ag balls; the first two contain primary Ag_3_Sn, the 3rd and 4th do not

Sn-5Ag solder balls containing large primary Ag_3_Sn plates had the typical microstructures overviewed previously in Fig. 2 (single-grain, beachball and interlaced), whereas Sn-5Ag balls with little (area fraction < 0.3%) to no primary Ag_3_Sn solidified with fully eutectic regions occupying most of the cross section. Figure 6a is an optical micrograph of a typical Sn-5Ag solder ball microstructure without primary Ag_3_Sn showing the whole solder ball, and Fig. 6b is a higher magnification micrograph of the boxed area in Fig. 6a. The entirety of the region in Fig. 6b is occupied by a β-Sn + Ag_3_Sn eutectic microstructure.

Figure 6c shows the EBSD mapping results from a typical Sn-5Ag solder ball that contains large fully eutectic regions. This ball is cyclic-twinned with a single ring similar to the beachball and interlaced microstructures in Fig. 2b–d.

Figure 6d is a column chart where the first column shows the percentage of solder balls with different solder microstructures among the Sn-5Ag solder balls that had noticeable primary Ag_3_Sn particles (such as the first two balls in Fig. 6f), and the second column shows the percentage among the balls that had little to no primary Ag_3_Sn particles in their cross sections (such as the 3rd and 4th balls in Fig. 6f). As primary Ag_3_Sn might exist in a solder ball but not in a cross section, the solder balls that did not have primary Ag_3_Sn in their cross section after the initial grinding were ground down gradually until either primary Ag_3_Sn particles appeared, or the solder balls were totally ground away. It is clear from Fig. 6d that the microstructure with fully eutectic regions only existed in solder balls with little to no primary Ag_3_Sn particles. Here, one out of 34 solder balls in the second column had an interlaced microstructure instead of the fully eutectic microstructure and, therefore, should be expected to belong to the first column, as it is probable that this one solder ball did contain a large primary Ag_3_Sn but it was ground away during the initial grinding, although this cannot be confirmed.

The key role played by primary Ag_3_Sn was to change the melt composition following the extended Ag_3_Sn liquidus line, such that β-Sn nucleated in liquid of lower Ag content in solder balls containing large primary Ag_3_Sn. To show this effect, in Fig. 6e, data points of Sn-5Ag balls with large Ag_3_Sn particles have been shifted onto the extended Ag_3_Sn liquidus line and data points of Sn-5Ag balls with little to no primary Ag_3_Sn remain at 5 wt% Ag. Note that for the solder balls with a small amount of primary Ag_3_Sn in the second column in Fig. 6d, those small primary Ag_3_Sn particles were always far away from the β-Sn nucleation location and, thus, they did not significantly affect the local Ag content in the liquid near the β-Sn nucleation point, which was considered to stay close to 5 wt%. The black diamond at 5 wt% Ag in Fig. 6e represents the previously mentioned fully interlaced outlier sample, which probably contains primary Ag_3_Sn and belong to the extended Ag_3_Sn liquidus line. The finding in Sn-5Ag that Ag_3_Sn nucleated before β-Sn in some balls and β-Sn nucleated first in other balls over a wide range of β-Sn nucleation undercoolings (Fig. 6e) is consistent with the study of Cui et al. [51] that identified nucleation difficulties for both phases and competitive nucleation between them in 500 μm diameter balls.

**Fig. 7:**
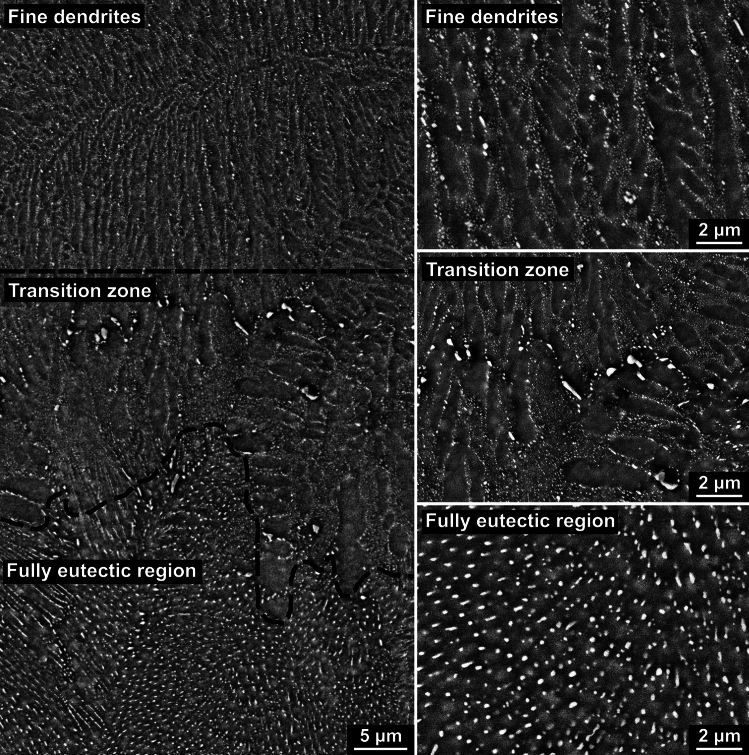
Typical microstructure of a Sn–5Ag solder ball with fully eutectic regions. The left image is a low magnification backscattered SEM image that shows the microstructure of a large area containing both the fine dendrite region and the fully eutectic region. The right images are high magnification SEM backscattered images that show the fine dendrites, the fully eutectic region, and the transition zone between them separately

It was observed that Sn-5Ag balls with fully eutectic regions always also contained small regions of β-Sn dendrites that were usually very fine with secondary dendrite arm spacing of < 1 *μ*m. Figure 7 shows an example. The left of Fig. 7 is a backscattered SEM image at lower magnification, containing a fine dendrite region, a fully eutectic region, and a transition zone in between. The transition zone contains coarser dendrites and some large Ag_3_Sn particles. It is believed that the coarser dendrites are due to the slower dendrite growth speed away from the nucleation point, which is within the fine dendrite region, as the interface temperature increased rapidly after β-Sn nucleation. The large Ag_3_Sn particles are probably from the preferred precipitation of Ag_3_Sn on the grain boundaries after solidification, as the transition zone always contains β-Sn grain boundaries, similar to ref [50] (e.g. Figure 11 in that paper).

The three images on the right of Fig. 7 are at higher magnification and exhibit the two microstructures and the transition zone individually. It can be concluded that the fine dendrites were the first microstructure that grew during recalescence after β-Sn nucleation, due to their very fine length scale and the fact that other microstructural features appeared to grow away from them.

**Fig. 8:**
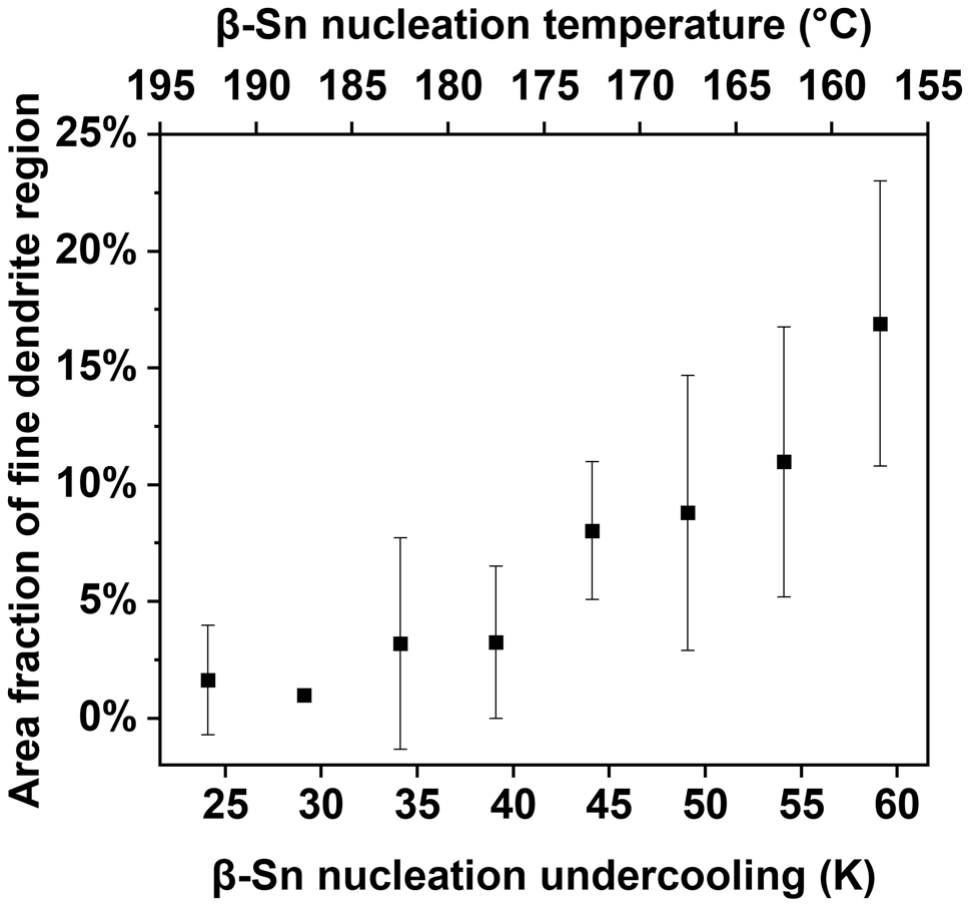
Scatter plot of average fine dendrite area fraction versus β-Sn nucleation undercooling for Sn–5Ag balls with large regions of fully eutectic microstructure (i.e. Sn–5Ag balls without large primary Ag_3_Sn). The error bar represents the standard deviation from all the measured balls in each undercooling bin

The fine dendrite microstructures were found to match with regions of slightly different β-Sn orientation from the main orientations which could be easily observed in polarized light images and EBSD maps. Therefore, these misoriented regions were used to measure the area fractions of fine dendrite regions. For all Sn-5Ag solder balls with fully eutectic and fine dendrite regions, such as that in Fig. 7, the area fraction of the fine dendrite regions in their cross sections was measured, yielding Fig. 8. There is a clear trend that when the undercooling was deeper, the fine dendrites occupied an increased volume fraction of the solder balls. This result is consistent with the very fine β-Sn dendrites growing during recalescence. In the simplified case of adiabatic conditions, the fraction solidified during recalescence is directly proportional to the increase of temperature during recalescence, ΔT_r_, as shown in (1), where c_p_ is the heat capacity for the liquid, L is the latent heat of fusion for β-Sn, and both are approximately constant.

1 $$\Delta f_{s} \, = \,\left( {\frac{{C_{p} }}{L}} \right) \cdot \Delta T_{r}$$

As will be discussed later, during recalescence, the fine dendrites grow until a certain temperature is reached, after which the fully eutectic microstructure starts growing. Therefore, deeper initial undercooling gives larger Δ*T*_r_ for the solidification of fine dendrites, leading to a larger volume fraction of fine dendrites.

### Solidification microstructure selection map

Although not reported before in small Sn-Ag solder balls, fully eutectic β-Sn + Ag_3_Sn microstructures have been observed in past work on unidirectional solidification of Sn-Ag alloys into a positive temperature gradient [22, 25, 52]. There, the fully eutectic microstructure is related to growth competition between β-Sn dendrite tips, a β-Sn + Ag_3_Sn eutectic front and primary Ag_3_Sn growth tips, and the competitive growth criterion is: for a given isotherm velocity, the microstructure that can grow at highest temperature is selected[14]. In the present study, the growth is into an undercooled melt and the competitive growth criterion is: for a given melt temperature, the microstructure that can grow at highest velocity is selected [14]. The relationship between the eutectic growth temperature, ${T}_{G}^{E}$, and growth velocity, V, for the β-Sn + Ag_3_Sn eutectic was determined by Hou et al. [25] in unidirectional solidification into a positive temperature gradient to be (2), which is in the form expected of Jackson-Hunt eutectic growth theory[53] and its extension to non-faceted—faceted (nf-f) eutectics[54]. The unit for ${T}_{G}^{E}$ and *V* in (2) are °C and *µ*m/s, respectively.

2 $${T}_{G}^{E}=221.5-0.44\sqrt{V}$$

The relationship between β-Sn dendrite growth velocity, *V*, and β-Sn dendrite growth undercooling, Δ*T*^Sn^, can be obtained from the Lipton-Glicksman-Kurz model (the LGK model) [55], which is a widely used model for dendrite growth into an undercooled alloy melt. (3) shows the fit of the LGK model for Sn–Ag within the range of undercooling and composition in this study:

3 $$\Delta {T}^{Sn}=28.6{C}_{0}^{0.59}{V}^{0.35},$$

where *C*_0_ is the Ag content in wt%. The units for *V* and Δ*T*^Sn^ are m s^−1^ and K, respectively. The procedure to obtain (3) is outlined in Appendix A along with a comparison between the LGK model, which assumes local interfacial equilibrium, and the combined model of Lipton-Kurz-Trivedi [56, 57] and Boettinger-Coriell-Trivedi [58] (the LKT-BCT model) which accounts for some rapid solidification effects. Appendix A shows that both models give similar results for the range of undercoolings relevant to the competition between β-Sn dendrites and a eutectic front in the composition range. The β-Sn dendrite growth temperature is then calculated with (4):

4 $$T_{G}^{{Sn}} \, = \,T_{{liq}} - \Delta T^{{Sn}}$$

where *T*_liq_ is the β-Sn liquidus temperature.

**Fig. 9:**
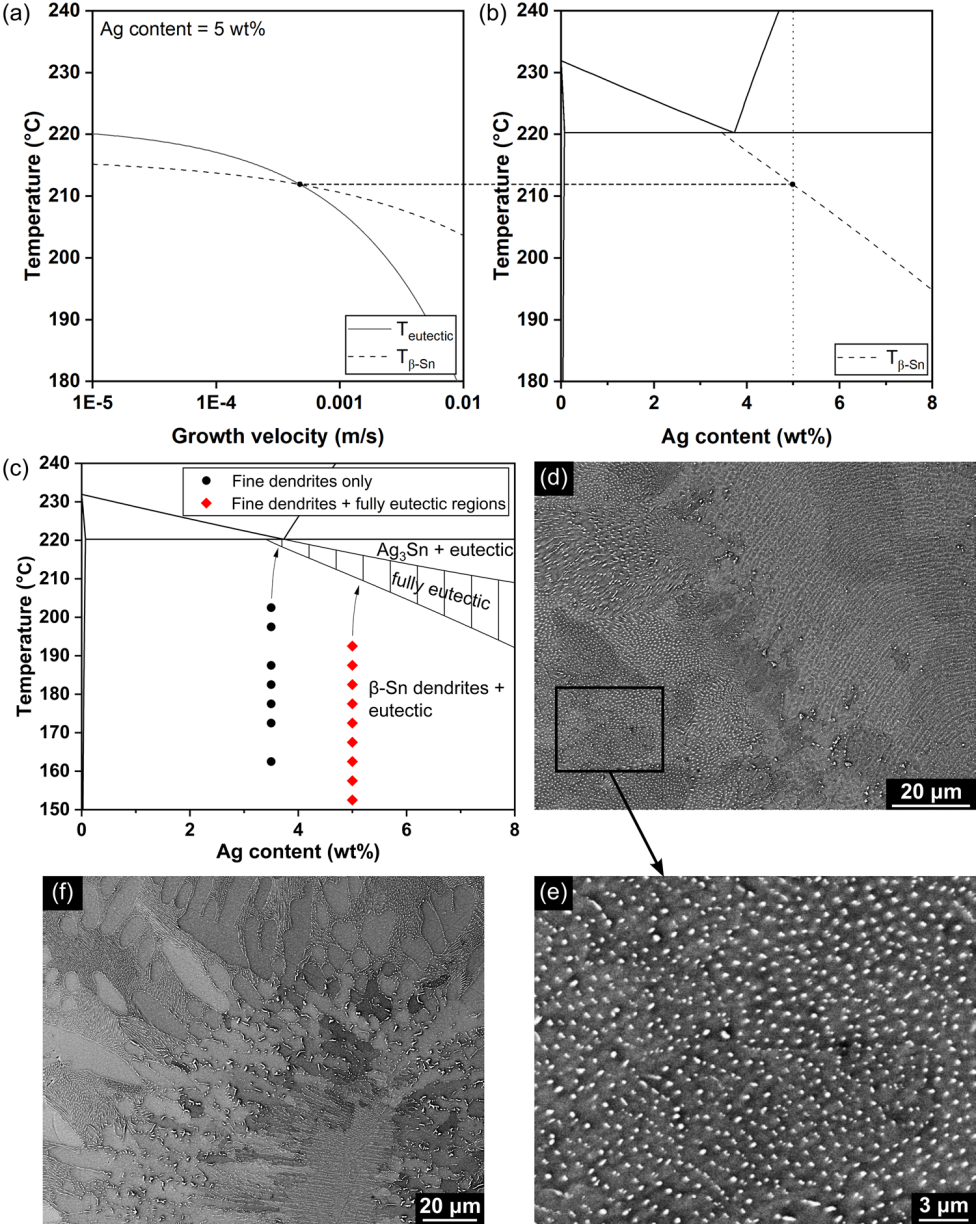
Study on the coupled zone for Sn-Ag alloy. **a** The curves of β-Sn and β-Sn + Ag_3_Sn eutectic growth temperatures versus the growth velocity when Ag content = 5 wt%. **b** The calculated coupled zone boundary on the Sn–Ag phase diagram. A dashed line is drawn to show how the coupled zone boundary at 5 wt% in **b** is calculated by the intersection in (**a**). **c** The completed coupled zone on the Sn–Ag phase diagram with the experimental data of Sn–3.5Ag and Sn–5Ag solder balls that contain fine dendrites marked on it. The two arrows indicate how the temperature—composition combination of the melt changes after the solidification starts. **d**, **e** Typical backscattered SEM images of Sn–5Ag solder balls that contain fine dendrites. **e** is the zoomed-in image of the highlighted area in (**d**). **f** Typical backscattered SEM image of Sn–3.5Ag solder balls that contain fine dendrites

The region where a fully eutectic microstructure grows is commonly called the coupled zone [14, 59]. Figure 9a, b illustrate the process to calculate the competitive growth and the coupled zone. Based on (2) and (4), Fig. 9a shows the relationship between the growth temperature and the growth velocity of β-Sn dendrite tips and a β-Sn + Ag_3_Sn eutectic front when the Ag content is 5 wt%. The two curves intersect at approximately 212 °C (i.e. the boundary temperature for Ag = 5 wt%). At temperatures below 212 °C, β-Sn dendrites can grow faster than a eutectic front, generating a microstructure of β-Sn dendrites with eutectic filling in the remaining liquid behind. At temperatures above 212 °C, a eutectic front grows fastest generating a fully eutectic microstructure. As ${T}_{G}^{Sn}$ is a function of Ag content (4), the boundary temperature changes with Ag content. The boundary temperatures at different Ag contents were calculated and the curve of boundary temperature versus Ag content was drawn as a dashed line in the Sn–Ag phase diagram in Fig. 9b.

In Fig. 9c, a second boundary line is drawn representing the transition to where primary Ag_3_Sn can grow at highest velocity which must also be present in the SMSM theoretically. However, due to a lack of knowledge of the kinetic growth undercooling for the faceted Ag_3_Sn plates, we did not calculate it here. Instead, an approximate primary Ag_3_Sn boundary is plotted in Fig. 9c as a straight line with slope slightly lower than the extended β-Sn liquidus line since the eutectic should grow beneath the extended liquidus of each solid phase. In Fig. 9c, the eutectic coupled zone is skewed towards the faceted Ag_3_Sn side as has been measured for the Sn–Ag_3_Sn eutectic in unidirectional solidification experiments [22, 25, 52], and as is common for nf-f eutectics [54]. The fully eutectic microstructure in the Sn–5Ag solder balls is expected to form when the solder ball temperature—composition combination is located in the coupled zone during solidification.

Data of the Sn-3.5Ag and Sn-5Ag solder balls that contained fine dendrites are marked in Fig. 9c with distinct labels and colours. With Fig. 9c, the formation of the microstructures in the Sn–3.5Ag and Sn–5Ag solder balls can be explained. Since Sn–3.5Ag and Sn–5Ag balls containing fine dendrites were always observed to have little to no primary Ag_3_Sn, the Ag contents in the melt were close to 3.5 wt% and 5 wt%, respectively, when β-Sn nucleated. At the moment of β-Sn nucleation, the temperature—composition combination of all balls of both alloys was located in the β-Sn dendrites + eutectic region in Fig. 9c. Thus, β-Sn dendrites formed first in both compositions. At these moderately deep undercoolings, dendrite growth was fast, leading to the fine dendritic microstructure. With the solidification of β-Sn dendrites, latent heat release quickly raised the temperature of the solder balls (Fig. 1d). As a result, the temperature—composition combination of the solder balls moved approximately following the two arrows in Fig. 9c. For Sn–5Ag, the temperature—composition combination entered the coupled zone and then the fully eutectic microstructure grew until the end of solidification, leading to a microstructure that contained a region of fine β-Sn dendrites and a larger region of fully eutectic microstructure, e.g. Figure 9d, e. For Sn–3.5Ag, the temperature—composition combination did not enter the coupled zone and, thus, the fully eutectic microstructure was not formed. Instead, there is a changing length scale of the β-Sn dendrites related to the changing undercooling during and after recalescence, leading to the microstructure of fine dendrites plus coarse dendrites shown in Fig. 9f.

**Fig. 10:**
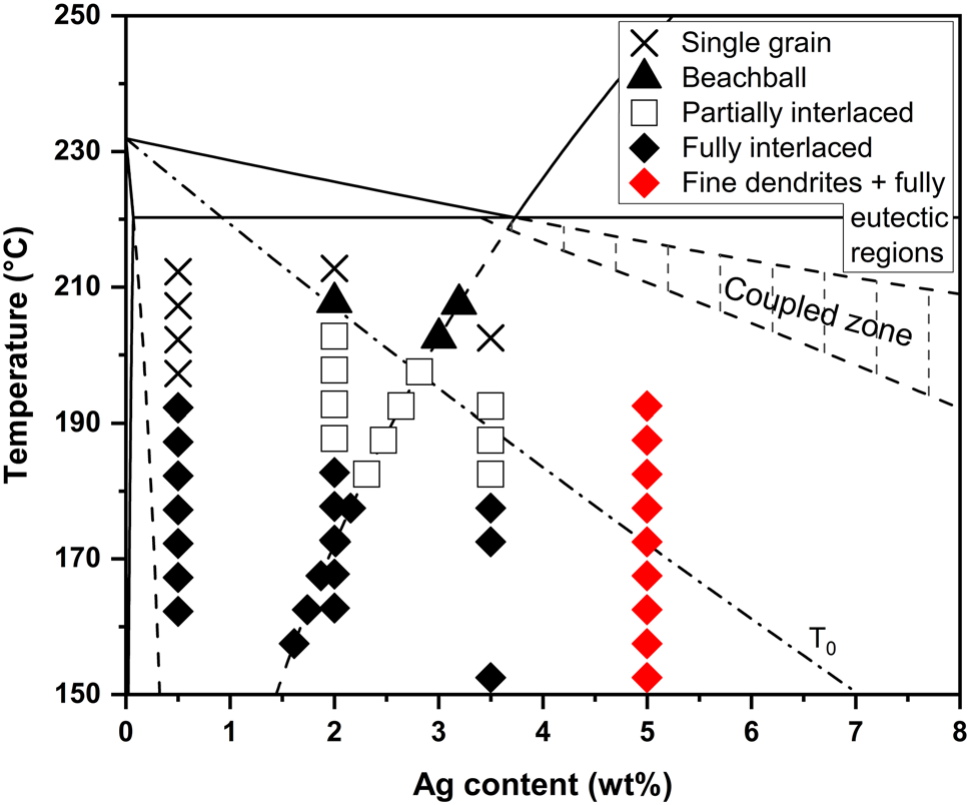
Solidification microstructure selection map (SMSM) for Sn–Ag solder balls. The phase diagram, metastable liquidus and solidus extensions and the T_0_ line were calculated in Thermo-Calc with the TCSLD4.1 database. Symbols show the most commonly occurring microstructure in each undercooling bin. Note, however, that three or four microstructure types commonly occurred within one undercooling bin for bins with black datapoints (Fig. 4). Balls containing large primary Ag_3_Sn have been shifted onto the extended Ag_3_Sn liquidus line

**Fig. 11:**
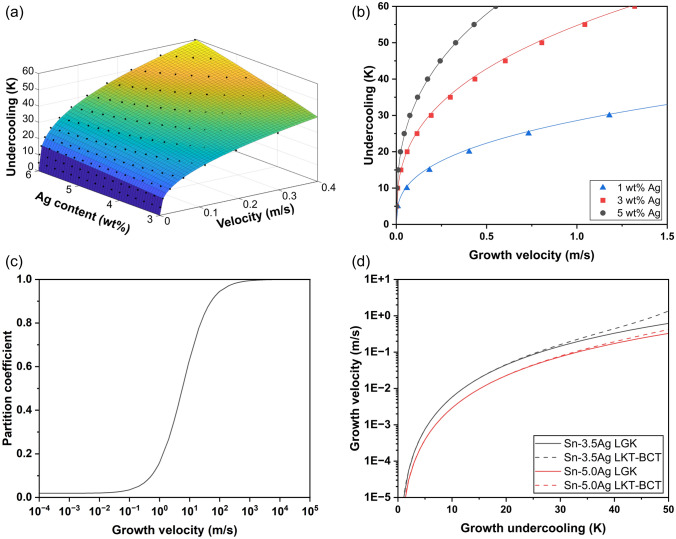
Analysis with the LGK and LKT-BCT models. **a** Fitting of the data calculated from the LGK model. The fitting equation is (3). **b** Comparison between the fitting equation and the data calculated from LGK at certain Ag contents. **c** Partition coefficient versus growth velocity curve for the Sn–Ag system. **d** Comparison between the growth velocity versus undercooling curves from the LGK model and those from the LKT-BCT model

Figure 10 is a Sn–Ag solidification microstructure selection map (SMSM) combining all parts of this study together. Each datapoint indicates the most commonly occurring microstructure in each bin for different combinations of β-Sn nucleation temperature and alloy composition. The Sn–3.5Ag and Sn–5Ag balls that contained large primary Ag_3_Sn particles have been shifted onto the extended Ag_3_Sn liquidus line to account for the changed melt compositions before β-Sn nucleation occurred. Please note that the black symbols show only the most commonly occurring microstructure and that, as discussed earlier with Fig. 4, it was common for three or four microstructure types to occur in many of the undercooling bins and there were no sharp transitions between the microstructures with black symbols.

In Fig. 10, we see three features discussed above: (i) Balls with liquid compositions of 1.6–3.5wt% Ag, including 3.5 and 5 wt% Ag balls shifted onto the extended Ag_3_Sn liquidus line by the prior growth of large primary Ag_3_Sn, have a dominant microstructure of primary β-Sn that changes from single-grain to beachball, partially interlaced and finally fully interlaced with deeper undercooling, as discussed with Fig. 4. (ii) In contrast, in Sn–0.5Ag balls, the dominant microstructure changes directly from single-grain to fully interlaced when the β-Sn nucleation undercooling > 35 K. This is presumably because decreasing the Ag concentration suppresses β-Sn cyclic twinning until a relatively deep undercooling is reached and, at such a deep nucleation undercooling, cyclic-twinned balls tend to have a fully interlaced microstructure, as discussed with Fig. 4f. (iii) In Sn-5Ag balls where Ag_3_Sn did not nucleate first (or where Ag_3_Sn forms only a very small volume fraction), the microstructure contains a small fraction of fine β-Sn dendrites that grew during recalescence and a large region of fully eutectic microstructure that grew once recalescence brought the composition and temperature into the coupled zone, as discussed with Fig. 9.

Figure 10 also shows that, in the majority of solder balls, nucleation occurred below the β-Sn T_0_ line. The T₀ line shows the composition-temperature combinations at which the Gibbs energies of the liquid and solid phases are equal. It is thermodynamically possible in these balls for the liquid to transform directly into β-Sn of the same composition during solidification. A kinetic analysis with the LKT-BCT model in Appendix A indicates that partial solute trapping is expected during recalescence in many of the balls in this study. The boundary between the temperature—composition combinations dominated by the beachball microstructure and those dominated by the partially interlaced microstructure is close to the T_0_ line. As interlaced regions appear to be near the nucleation point (e.g. Figure 5c) [6, 60], and are thus related to the microstructure that grew first, this indicates that the interlaced microstructure may have been affected by solute trapping and the subsequent transformation of supersaturated β-Sn. However, Fig. 10 only shows the majority microstructure in each undercooling bin and some balls had a partially interlaced microstructure at much smaller undercooling. Thus, from these experiments, no simple relationship is revealed between partial solute trapping and microstructure, including interlacing.

The microstructure selection map in Fig. 10 provides a guide for future research on solder alloy design and microstructure control. Specifically, it shows how the microstructure could be tailored if the nucleation of β-Sn, Ag_3_Sn and the occurrence of cyclic twinning could each be controlled. For example, if Ag_3_Sn nucleation could be reproducibly suppressed, nearly fully eutectic microstructures could be achieved in Sn–5Ag solder balls. This would likely improve thermal fatigue performance which increases with increasing β-Sn + Ag_3_Sn eutectic fraction [61, 62]. Alternatively, to obtain Sn–Ag joints with fully interlaced microstructures, this study indicates that eutectic or hypoeutectic liquid compositions and bulk undercoolings of > 30 K are required. Fully interlaced microstructures have been identified as more desirable than beachball microstructures since they increase shear and creep strength [63, 64] and give better thermal fatigue resistance [65, 66] by relieving damage localization [67]. To promote fully interlaced microstructures, developing methods to suppress β-Sn nucleation (and suppress Ag_3_Sn nucleation to prevent large Ag_3_Sn plates) can be beneficial. There may be cases where single-grain joints are desired since these have been identified as giving better thermal fatigue performance than beachball joints because thermal fatigue damage localises at beachball boundaries [2]. For single-grain joints, methods would need to be developed to suppress cyclic twinning at/near the nucleation stage.

## Conclusions

A systematic study has been conducted, solidifying 500 *μ*m diameter Sn–Ag solder balls at an imposed cooling rate of 0.33 K s^−1^ for alloy compositions in the range 0.5–5.0 wt. % Ag. The resulting β-Sn nucleation undercoolings were in the range 10–70 K for each composition, enabling a direct study of the combined effects of Ag and undercooling on the microstructure of Sn-Ag solder balls. The results have been combined into a solidification microstructure selection map (SMSM) for Sn–Ag solder balls in composition—nucleation temperature space. The following new insights have emerged:

β-Sn cyclic twinning was promoted by deeper nucleation undercooling and by increased Ag concentration. The transition from single-grain to cyclic-twinned microstructures was not abrupt at a critical undercooling but occurred gradually over multiple undercooling bins, indicating an increasing probability of cyclic twinning with deepening undercooling.

In cyclic-twinned balls, deeper nucleation undercooling resulted in a gradual transition in the primary β-Sn morphology from beachball to partially interlaced and then fully interlaced microstructures, with a gradually increasing area fraction of interlacing.

The competition between the nucleation of Sn and Ag_3_Sn played a strong role in microstructure formation in undercooled melts, and particularly at compositions near the eutectic point. When Ag_3_Sn nucleated first, its growth changed the remaining liquid composition such that Sn nucleated in hypoeutectic liquid and subsequent solidification proceeded similar to lower-Ag alloys. When Sn nucleated first in Sn–5Ag, a small fraction of fine β-Sn dendrites grew first and the remaining microstructure was fully eutectic.

The solidification microstructure selection map (SMSM) is in reasonable agreement with coupled zone theory, accounting for whether Ag_3_Sn or Sn nucleate first in competitive nucleation.

Calculations of dendrite growth in undercooled Sn–Ag indicate that partial solute trapping is expected during recalescence at the deeper undercoolings measured in this study. However, no simple relationship was found between partial solute trapping and the microstructure transitions studied here.

The present study demonstrates how modifying the composition, controlling the β-Sn nucleation undercooling and suppressing the nucleation of Ag3Sn can affect the microstructure of Sn–Ag solders and as a result, their performance. More broadly, with this work as a fundamental guide, microstructure control could become an additional approach to designing next-generation lead-free solders.

## Electronic supplementary material

Below is the link to the electronic supplementary material.

Supplementary file1 (PDF 528 KB)

## Data Availability

The data in this work are available upon reasonable request.

## Appendix A

To model the relationship between the melt undercooling and dendrite tip growth velocity for the range of measured undercoolings (< 70 K), two models were considered. First, the LGK model [55] was utilised, which uses Ivantsov’s solution to the diffusion field around a dendrite tip [68, 69] and a marginal stability criterion [70–72]. In the LGK model, the dendrite tip growth undercooling is the sum of three undercoolings as given in (5), whose symbols, as well as the symbols from the following equations, are defined in Table

**Table 1:** The thermo-physical properties of the Sn-Ag system used in the LGK and LKT-BCT calculation. TC = Thermo-Calc TCSLD 4.1 database

Property	Symbol	Unit	Value	Ref
Dendrite tip growth velocity	V			
Dendrite tip radius	R			
Ag content	C_0_			
Growth undercooling	ΔT_g_			
Thermal Péclet number	P_t_	–		
Solutal Péclet number	P_c_	–		
Partition coefficient	k	–		
β-Sn liquidus slope	m’			
Interfacial kinetic coefficient	µ			
Thermal stability function	ξ_t_	–		
Solutal stability function	ξ_c_	–		
Equilibrium β-Sn liquidus temperature	T_L_	K		
Specific heat	c_p_	J/(kg∙K)	249	TC
Latent heat of fusion for β-Sn	L	J/kg	61,810.62	TC
Equilibrium β-Sn liquidus slope	m	K/wt pct	−3.14	TC
Equilibrium partition coefficient	k_0_	–	0.0191	TC
Gibbs–Thomson coefficient	Γ	m∙K	8.54 × 10^–8^	[73]
Solute diffusivity in the liquid	D	m^2^/s	1.82 × 10^–9^	[74]
Thermal diffusivity in the liquid	α	m^2^/s	1.5 × 10^–5^	[75]
Interatomic distance in Sn	a_0_	m	3.07 × 10^–10^	[76]
Speed of sound in the liquid	V_0_	m/s	2.47 × 10^3^	[77]
Stability constant	σ*	–	1/4π^2^	
Gas constant	R_0_	J/(mol∙K)	8.314	

^1^.

5 $$\Delta T_{g} = \frac{L}{{c_{p} }}Iv\left( {P_{t} } \right) + mC_{0} \left\{ {1 - \frac{1}{{1 - \left( {1 - k_{0} } \right)Iv\left( {P_{c} } \right)}}} \right\} + \frac{2\Gamma }{R}$$

The terms on the right-hand side are, from left to right, the thermal undercooling, $\Delta {T}_{t}$, the solutal undercooling Δ*T*_c_, and the curvature undercooling Δ*T*_r_. In (5), *P*_*t*_ and *P*_c_ are the thermal Peclet number and solutal Peclet number, respectively, and Iv(P_x_) is the Ivantsov function for each Peclet number:

6 $$\begin{array}{c}{P}_{t}=\frac{VR}{2\alpha }\end{array}$$

7 $$\begin{array}{c}{P}_{c}=\frac{VR}{2D}\end{array}$$

8 $${Iv(P}_{x})={P}_{x}\text{exp}({P}_{x}){E}_{1}({P}_{x})$$

In the LGK model, the dendrite tip radius is described with (9), which is obtained from the stability criterion [72].

9 $$R = \frac{{{\raise0.7ex\hbox{$\Gamma $} \!\mathord{\left/ {\vphantom {\Gamma {\sigma^{*} }}}\right.\kern-0pt} \!\lower0.7ex\hbox{${\sigma^{*} }$}}}}{{\frac{{P_{t} L}}{{c_{p} }} - \frac{{P_{c} mC_{0} \left( {1 - k_{0} } \right)}}{{1 - \left( {1 - k_{0} } \right)Iv\left( {P_{c} } \right)}}}}$$

With (5) and (9), under a certain combination of Δ*T*_g_ and *C*_0_, the growth velocity, *V*, and dendrite tip radius, *R*, can be calculated using iterative numerical methods, such as the 2D Newton–Raphson method. The calculated combinations of undercooling, growth velocity and Ag content are plotted as scatter points in Fig. 11a. The fit of the scatter points is then calculated to be (3), which gives the coloured surface in Fig. 11a. The fit to the calculated data are further visualised in Fig. 11b.

The model of Lipton et al. [56, 57] and Boettinger et al. [58] (the LKT-BCT model) applies corrections to the LGK model [55] to extend it to be valid within the framework of rapid solidification [15]. In the LKT-BCT model, a kinetic undercooling is introduced that contributes to the growth undercooling, as shown in (10):

10 $$\Delta T_{g} = \frac{L}{{c_{p} }}Iv\left( {P_{t} } \right) + mC_{0} \left\{ {1 - \frac{{m^{\prime}/m}}{{1 - \left( {1 - k_{0} } \right)Iv\left( {P_{c} } \right)}}} \right\} + \frac{2\Gamma }{R}\, + \,\frac{V}{\mu },$$

where the last term on the right-hand side accounts for the kinetic undercooling ΔT_k_.

The velocity-dependant partition coefficient k and liquidus slope m’ in (10) are given by:

11 $$k=\frac{{k}_{0}+\left({a}_{0}V/D\right)}{1+\left({a}_{0}V/D\right)}$$

12 $$m^{\prime} = m\left\{ {1\, + \,\frac{{k_{0} - k\left[ {1 - {\text{ln}}\left( {k/k_{0} } \right)} \right]}}{{1 - k_{0} }}} \right\}$$

Eq. (11) is plotted out in Fig. 11c for the Sn–Ag system. The interfacial kinetic coefficient *μ* in (10) is defined as:

13 $$\mu =\frac{L{V}_{0}}{{R}_{0}{T}_{L}^{2}}$$

The dendrite tip radius in the LKT-BCT model is:

14 $$R = \frac{{{\raise0.7ex\hbox{$\Gamma $} \!\mathord{\left/ {\vphantom {\Gamma {\sigma^{*} }}}\right.\kern-0pt} \!\lower0.7ex\hbox{${\sigma^{*} }$}}}}{{\frac{{\xi_{t} P_{t} L}}{{c_{p} }} - \frac{{2\xi_{c} P_{c} m^{\prime}C_{0} \left( {1 - k} \right)}}{{1 - \left( {1 - k} \right)Iv\left( {P_{c} } \right)}}}},$$

where the stability functions ξ_t_ and ξ_c_ are added compared to the LGK model:

15 $$\xi_{t} = 1 - \frac{1}{{\sqrt {1 + 1/\left( {\sigma^{*} P_{t}^{2} } \right)} }}$$

16 $$\xi_{c} = 1 + \frac{2k}{{1 - 2k - \sqrt {1 + 1/\left( {\sigma^{*} P_{c}^{2} } \right)} }}$$

Similar to the LGK model, iterative numerical methods were used to calculate the results of the LKT-BCT method. The curves of β-Sn dendrite growth velocity versus β-Sn growth undercooling for both the LGK and the LKT-BCT models are plotted in Fig. 11d. Based on Fig. 11d, in the relevant composition and undercooling ranges for the coupled zone calculation, both models give similar results, and the application of the relatively simpler LGK model is justified for the coupled zone calculation in Fig. 9c.
