# Supplementary material for: Effects of Ag and melt undercooling on the microstructure of Sn–Ag solder balls
Source: J Mater Sci Mater Electron. 2025 Jun 2;36(16):942. doi: 10.1007/s10854-025-14979-6 (PMC12130131; doi:10.1007/s10854-025-14979-6)
Supplement: Supplementary file 1 — Supplementary file1 (PDF 528 KB) [file 10854_2025_14979_MOESM1_ESM.pdf]

Supplementary Information for ‘Effects of Ag and melt undercooling on the microstructure of Sn-Ag solder balls’ on Journal of Materials Science: Materials in Electronics

Sihan Sun[a,*], Ao Li[a], Chao Cheng[a], Christopher M. Gourlay[a, b]

[a] Department of Materials, Imperial College London, London. SW7 2AZ. UK

* Corresponding author. Email address: sihan.sun16@imperial.ac.uk

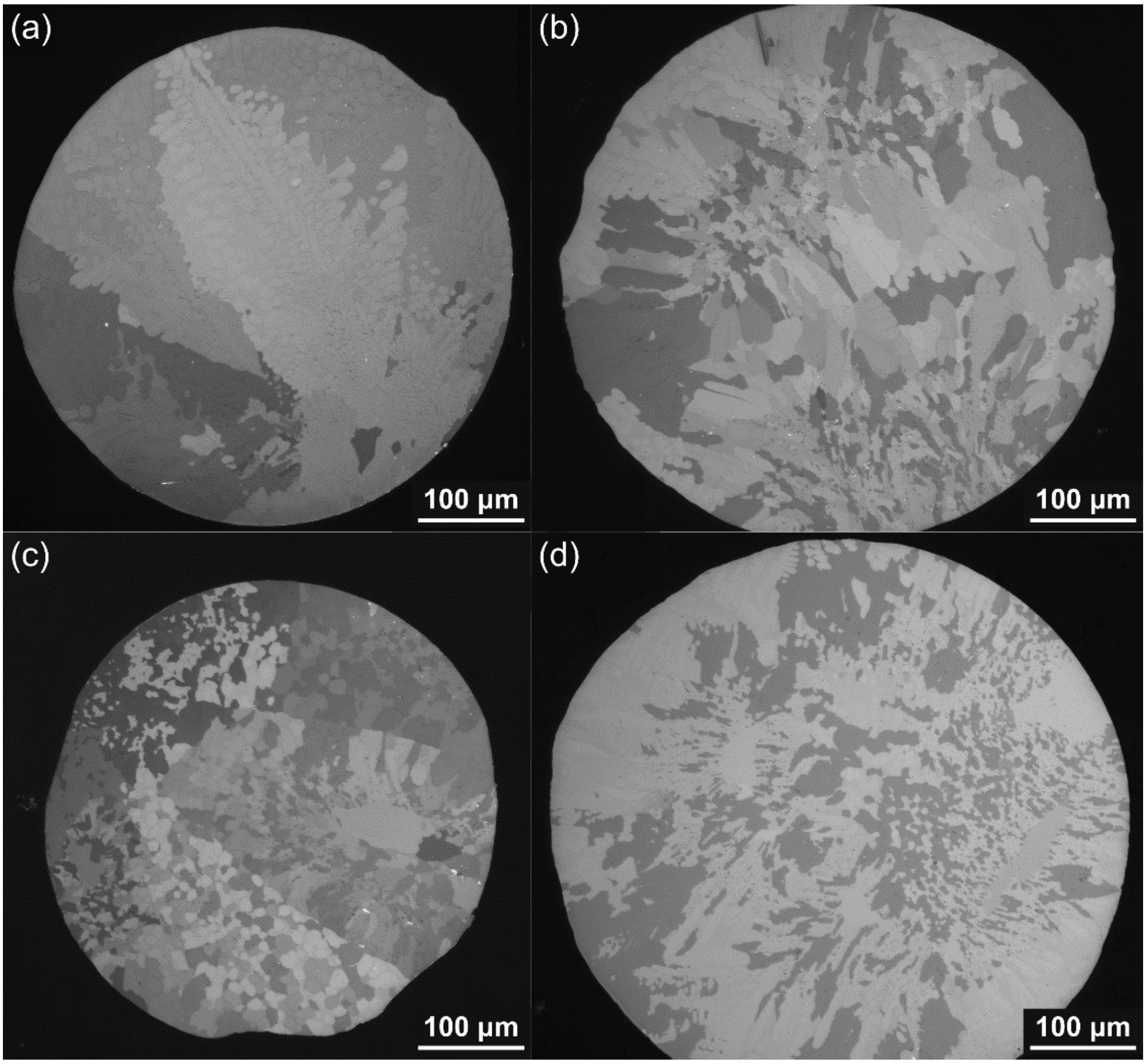

Figure S1. Polarized optical micrographs of the four Sn-3.5Ag balls that are not included in Figure 5.

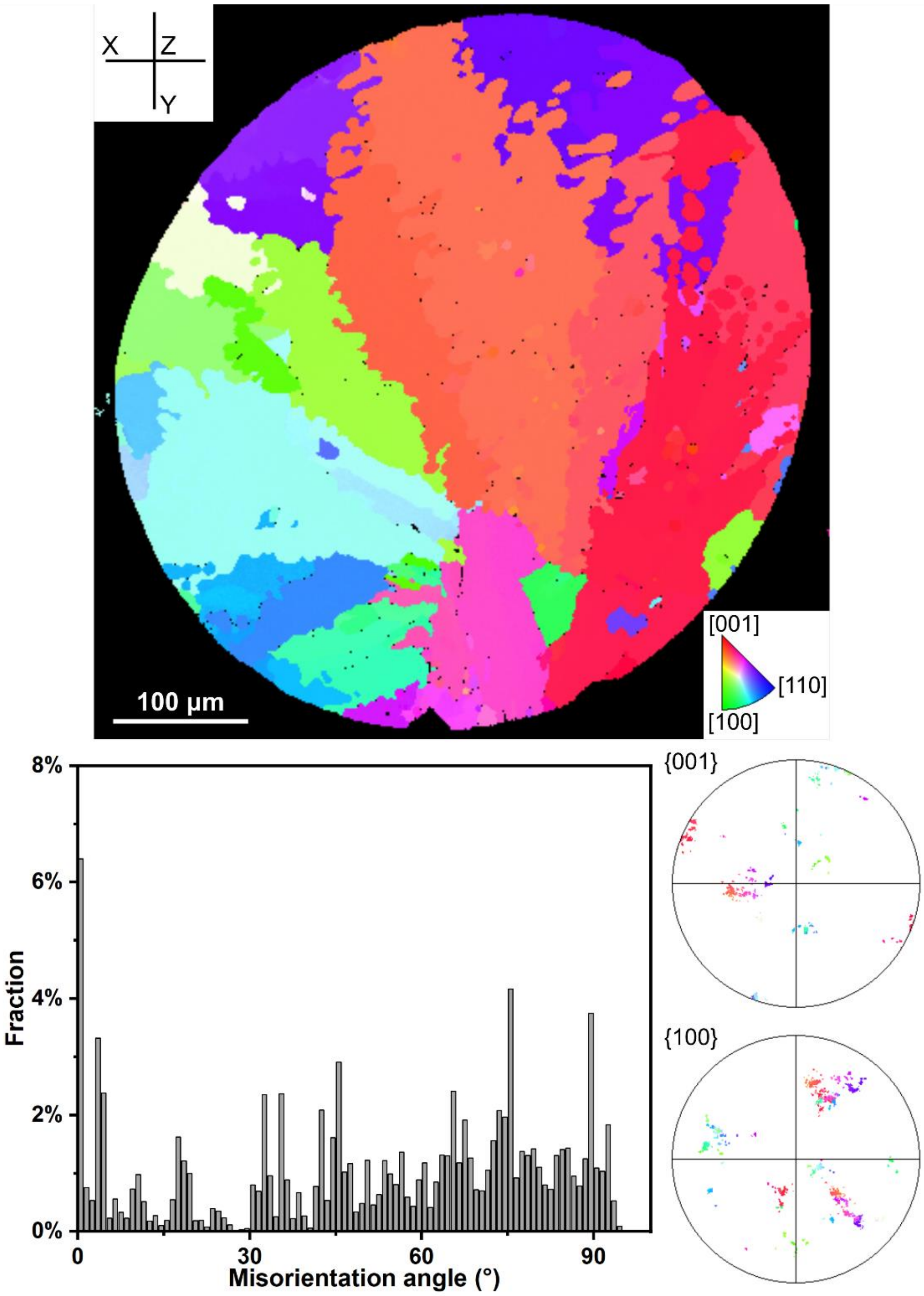

Figure S2. EBSD orientation map (IPF-X), misorientation distribution chart and {001}, {100} pole figures of the solder ball in Figure S1(a).
